# Immune-Mediated Diseases Following COVID-19 Vaccination: Report of a Teaching Hospital-Based Case-Series

**DOI:** 10.3390/jcm11247484

**Published:** 2022-12-16

**Authors:** Eric Liozon, Matthieu Filloux, Simon Parreau, Guillaume Gondran, Holy Bezanahary, Kim-Heang Ly, Anne-Laure Fauchais

**Affiliations:** 1 Departments of Internal Medicine, Dupuytren University Hospital, 87042 Limoges, France; 2 Department of Immunology and Immunogenetics, University Hospital of Limoges, 87042 Limoges, France

**Keywords:** COVID-19, vaccination, side, immune-mediated, giant cell arteritis, HLA-DR

## Abstract

The occurrence and course of immune-mediated diseases (IMDs) following COVID-19 vaccination has been little explored so far. We retrieved, among adult patients hospitalized at the Internal Department of a French university hospital up to May 2022, all those who had developed, or relapsed to, an IMD less than 3 weeks following COVID-19 vaccination, without other triggers. Twenty-seven (24 new-onset) post-COVID-19 vaccine IMDs were recorded. They comprised giant cell arteritis or polymyalgia rheumatica (*n* = 16, HLA-DRB1*04 in 58% of 12 assessed GCA cases), immune-mediated necrotizing myositis or acute rhabdomyolysis, systemic vasculitis, immune thrombocytopenic purpura, rheumatoid arthritis, anti-synthetase syndrome, and adult-onset Still’s disease. The causative vaccines were mRNA-based (20 cases) or viral vector-based (7 cases). The IMD typically occurred after the first vaccine dose, with an average delay of 8 (5 SD) days. The patients’ mean age was 67 years, and 58% were women. The IMDs had protracted courses in all but three of the patients and typically required high-dose glucocorticoids, in combination with immunomodulators in 13 patients. One patient died of intractable rhabdomyolysis, whereas five suffered permanent damage from IMDs. Eleven patients with well-controlled IMDs completed their COVID-19 vaccination schedule, and two suffered mild IMD relapses. There is a risk of IMDs, notably GCA/PMR, and muscle disorders, following COVID-19 vaccination. Such adverse reactions typically occurred after the first dose, raising concern about subsequent COVID-19 vaccinations. However, early re-challenge in well-controlled IMDs appeared safe.

## 1. Introduction

The speed and severe impact of the coronavirus disease 2019 (COVID-19) pandemic led to rapid development of vaccines. Phase-II trials have shown reassuring safety profiles in mRNA-based vaccines and viral vector-based vaccines [1,2,3]. Likewise, cross-sectional or longitudinal studies on the safety of several COVID-19 vaccines in autoimmune inflammatory rheumatic diseases did not detect a risk signal [4,5]. Nevertheless, recent safety data from population-wide surveys have suggested significant side effects, notably severe thrombotic events via vaccine-induced immune thrombotic thrombocytopenia [6]. Although vaccines are linked to several immune-mediated diseases (IMDs) [7,8,9,10,11,12,13], such reports on COVID-19 vaccines are limited, beside single case reports, to a few case-series [14,15,16,17]. Therefore, we evaluated the occurrence, or flare-up, of IMDs reported in the Internal Medicine Department of a University Hospital during the period of mass vaccination against COVID-19.

## 2. Methods

### 2.1. Study Design

Starting in January 2021, when COVID-19 vaccination became progressively available, we retrieved all reports of IMDs occurring after vaccination among consecutively hospitalized patients hospitalized in the Internal Medicine Department of a University Hospital. The catchment area of the hospital roughly corresponds to the population of the Limousin region, e.g., 723,784 people, 436,360 being over 40 years of age. As of 31 May 2022, at study termination, 82% of the regional population were vaccinated at least once against severe acute respiratory syndrome coronavirus-2 (SARS-CoV-2). However, precise calculation of the regional incidence of post-COVID-19 vaccine IMDs cannot be obtained, since it is expected that not all patients with an IMD will be referred, or reported, to the regional hospital. Details regarding the demographic information, significant comorbid conditions, date of diagnosis, clinical features, biological abnormalities at disease onset, use of glucocorticoid treatments including pulse methylprednisolone and/or prednisone, use and type of steroid-sparing agents, relapses during prednisone tapering, survival, recovery from IMD, and total duration of treatment were retrieved for each patient. We also aim to assess the influence of HLA-DRB1/DQB1 polymorphism on COVID-19 vaccine-induced IMDs, in particular the frequency of HLA-DRB1*01 and DRB1*04 alleles. Both alleles are risk factors for autoimmune/auto-inflammatory syndrome induced by adjuvants (ASIA) [8].

### 2.2. Case Series

*Inclusion criteria*: The case series comprised cases of giant cell arteritis (GCA), polymyalgia rheumatica (PMR), immune-mediated necrotizing myositis (IMNM) or acute rhabdomyolysis, ANCA-associated vasculitis (AAV), Henoch-Shönlein purpura (HSP), adult-onset Still’s disease (AOSD), rheumatoid arthritis (RA), and idiopathic thrombocytopenic purpura (ITP) following COVID-19 vaccination. The inclusion criteria were (a) IMD diagnosed using currently accepted criteria. GCA was diagnosed based on the criteria of the American College of Rheumatology [18]; negative biopsy cases were regarded as true GCA cases provided there was a dramatic and sustained response to corticosteroid treatment. PMR was diagnosed using the 2012 EULAR/ACR criteria [19]. Rheumatoid arthritis was diagnosed using the 2010 EULAR/ACR classification criteria [20]. IMNM was characterized by proximal muscle weakness, a high serum creatine kinase level, widespread inflammatory muscle involvement on magnetic resonance imaging, myofiber necrosis with minimal inflammatory cell infiltrate on muscle biopsy, and no or little extra-muscular involvement, with or without specific autoantibodies in serum [21]. Diagnosis of the one case of eosinophilic granulomatosis with polyangiitis (EGPA) was based on the patient’s history of allergic asthma, blood eosinophilia 3.6 × 10^9^/L, multiple mononeuropathy, and ANCA positivity (anti-myeloperoxydase 49 UI/L). In this patient, peripheral nerve biopsy was considered unnecessary. In a patient with a prior diagnosis of AOSD that met most of Yamaguchi’s criteria [22], a late relapse following vaccination was ascertained by a flare-up of arthritis in both wrists and knees together with increased CRP and ferritin levels. ITP was defined as a platelet count persistently <100 G/L with exclusion of other causes of thrombocytopenia. Diagnosis of Henoch-Shönlein purpura (HSP) was based on the presence of at least three of the criteria of the American College of Rheumatology [23]. (b) No symptom or sign suggesting an IMD before vaccination. (c) The interval between vaccination and IMD onset did not exceed 3 weeks (in order to minimize the play of chance). (d) No other trigger (viral including current or recent SARS-CoV-2 or bacterial infection, or inciting drug) was evident. If a patient had been vaccinated at least twice (i.e., COVID-19 and influenza vaccination) within 3 weeks of IMD onset, responsibility was arbitrarily assigned to the vaccine received closest to IMD onset. (e) No spontaneous improvement of IMD; immunosuppressive/immunomodulatory treatment was required. Written informed consent was obtained from each patient. 

*HLA-DRB1/DQB1 genotyping*: HLA-DRB1/DQB1 genotyping was conducted using peripheral blood samples in 20 patients. To assess the presence of COVID-19 vaccine-associated HLA specificities in the subset of GCA/PMR, we compared the results of HLA-DR typing of 58 patients, comprising 14 patients with COVID-19 vaccine-induced GCA or PMR, 11 with other vaccine-induced (influenza vaccine, or pneumococcal vaccine) GCA, 17 with familial aggregation of GCA or PMR and, a random sample of 16 patients with GCA (e.g., without a vaccine trigger or familial aggregation). Familial cases occurring after a vaccination were arbitrarily included in the corresponding post-vaccination group.

## 3. Results

**Figure 1:**
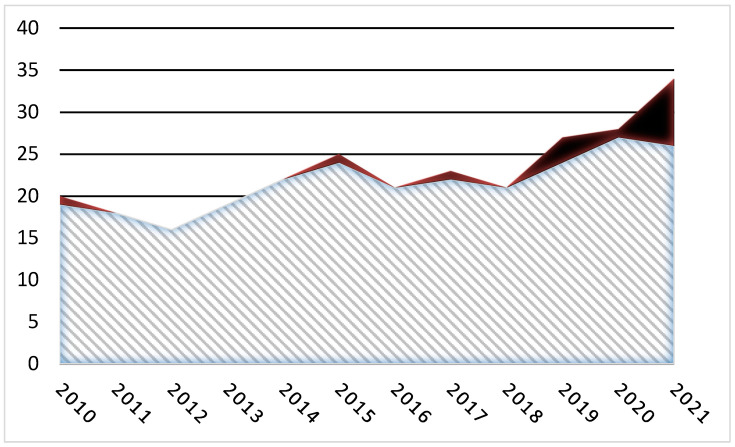
Numbers of patients with newly diagnosed GCA, including usual GCA and post-vaccine GCA, from 1 January 2010 to 31 December 2021. Gray shading, numbers of typical (e.g., without a vaccine trigger) GCA cases; black, post-vaccine cases.

*Characteristics of the cohort*: Thirty-seven reports were scrutinized for a possible relationship between IMD onset and vaccination. Three reports were excluded due to inconsistent temporal relationships. Four patients (Sjögren’s syndrome, systemic lupus, sarcoidosis, PMR), who reported self-limited inflammatory musculoskeletal or dermatological (sarcoidosis) manifestations soon after vaccination, were also excluded. Thirty post-vaccine disorders qualifying as IMDs were recorded within the study timeframe, 27 of which (90%; 22 new onset, 4 relapses) occurred after COVID-19 vaccination. These comprised 12 cases of GCA, four of PMR (one relapse), three of necrotizing myositis or acute rhabdomyolysis, three of systemic vasculitis (one EGPA, two SHP), two of ITP, and one each of relapsed rheumatoid arthritis, relapsed AOSD, and PL7-positive anti-synthetase syndrome with interstitial lung disease (ILD). Three other vaccine-induced IMDs were diagnosed within the study timeframe—two cases of GCA following seasonal influenza vaccination and a PMR following a second pneumococcal vaccination. Table 1 summarizes the demographics, diagnoses, COVID-19 vaccines, treatments, and outcomes of the patients. The patients’ mean age was 67 years (50–90 years), and 15 (56%) were women. The causative vaccines were ChAdOx1 (Vaxzevria^®^, AstraZeneca, Oxford, UK), BNT162b2 (Comirnaty^®^, Pfizer-BioNTech, New York, NY, USA), and mRNA-1273 (Spikevax^®^, Moderna, Cambridge, MA, USA) in 7, 18, and 2 patients, respectively. The IMD started after a first vaccine dose in 14 patients (52%), a second dose in seven patients (26%), and a third (booster) dose in six patients (22%). All three patients with acute IMNM, or rhabdomyolysis, had a negative or normal workup including serologic tests for HIV, herpes viruses, HTLV1 + 2, hepatitis A, B, and C, EBV, CMV, parvovirus, serum protein electrophoresis, antinuclear antibody test, anti-ds-DNA, ENA panel, DOT-myositis, C3, C4, rheumatoid factor, and ANCA. They all had widespread muscle inflammation with edema on MRI and a muscle biopsy demonstrating IMNM. Of the 12 GCA cases, nine were biopsy-proven. The delay between vaccination and GCA onset averaged 6 days (2–20 days). Three patients had permanent visual loss (two at diagnosis, one during an early relapse), bilaterally in one. All but three of the patients presented with an ESR > 60 mm/h (mean 92 mm/h) and a CRP level > 7 mg/dL (mean 12.3 mg/dL). The incidence of GCA in 2021 was higher than expected even after taking account of an upward trend (Figure 1).

*Precipitating/etiologic factors*: A potential etiologic factor or trigger, other than COVID-19 vaccination, was detected twice. A patient on statin for 3 years without muscle problems before receiving a booster dose of BNT162b2 (Case 19), tested positive for serum anti-HMG-CoA reductase antibody. One patient (Case 9) received seasonal influenza vaccination without early side effects, and 1 week later a booster dose of BNT162b2. In this patient, the systemic GCA began 6 days after COVID-19 vaccination.

*HLA-DRB1/DQB1 typing*: The most frequent HLA-DRB1 alleles were DRB1*04 (eight patients) and DRB1*11 (seven patients), whereas only three patients had HLA-DRB1*01. Most HLA-DQB1 alleles were DQB1*03 (14 patients, including 12 with GCA or PMR) and DQB1*02 (7 patients) (Table 2). No patients were positive for HLA-DQB1*01. Notably, 7 of 12 (58%) GCA patients, including both familial cases, had DRB1*04 alleles. There was no significant difference in the frequencies of HLA-DRB1 alleles among GCA/PMR subsets, and HLA-DRB1*04 was the most frequent allele (54%) (Table 3).

*Treatments and outcomes*: De-challenge (i.e., spontaneous remission or short-lived treatment without relapse) was demonstrated in three patients—two with IMNM (Cases 18 and 19) and one with ITP (Case 25). All but one of the patients (minor relapse of childhood-onset HSP) received first-line, often high-dose, glucocorticoid treatment, and 13 (50%) underwent additional treatments. All the GCA patients received high-dose glucocorticoid treatment, and most are currently undergoing tapering. One patient achieved remission after a 9-month prednisone course. Additional weekly subcutaneous tocilizumab was required for five GCA or PMR patients. One patient died from intractable rhabdomyolysis, despite intensive immunosuppressive treatment, whereas four patients (15%) had permanent damage from GCA (bilateral blindness), EGPA (bilateral median nerve palsy), or antisynthetase syndrome (restrictive lung disease). Twelve of sixteen eligible patients agreed to complete their COVID-19 vaccination during a quiescent phase of IMD, among whom 11 did so (crossover with an mRNA-based vaccine for two patients). Only two re-challenged patients experienced a disease flare—one GCA patient (Case 1) who reported a self-limited episode of polymyalgia rheumatica after the second vaccination and the patient with EGPA (Case 20), who experienced a mild flare of multiple mononeuropathy 3 days following a fourth mRNA-based (mRNA-1273, Moderna^®^) vaccine dose.

## 4. Discussion

### 4.1. Relationship between COVID-19 Vaccines and Subsequent IMDs

Vaccine-induced IMDs—including GCA, PMR, IMNM, AAV, HSP, AOSD, and ITP—have long been described but are rare [8,9,10,11,12,13]. We report herein a series of incident cases of IMDs following COVID-19 vaccination, recruited in the Internal Medicine Department of our University Hospital during the period of widespread vaccination. Some other trigger was present only in two patients. In these two patients (GCA, IMNM), the first symptoms of IMD occurred less than 1 week after COVID-19 vaccination. A causative relationship between vaccination and subsequent IMD is conjectural in the 27 patients, although re-challenge in two patients (Cases 1 and 20) resulted in a limited disease flare, reinforcing the hypothesis of a causal relationship between COVID-19 vaccination and GCA. The nine other re-challenged patients completed their vaccination schedule, up to two additional shots, without experiencing disease relapse. Significantly, re-vaccination was attempted during a quiescent phase of treated illness in these patients, which may have prevented them from disease flare-up. The patient with AOSD (Case 24) received her first two vaccine shots during treatment with low-dose methotrexate, without harm, and the offending booster shot while untreated for 4 months. Finally, de-challenge was rare (i.e., one in eight patients). Therefore, a causal relationship between COVID-19 vaccination and subsequent IMDs is not supported by our data. Nevertheless, the strong temporal relationship between the two events raises legitimate safety concerns about COVID-19 vaccines in these patients.

### 4.2. Incidence of IMDs after COVID-19 Vaccination

Although the regional IMD incidence following COVID-19 vaccination could not be calculated from our hospital-based recruitment, it might be not negligible, especially in individuals over 50 years of age. In a study of neurological IMDs occurring within 4 weeks of SARS-CoV-2 vaccination, the population-based incidence was 1/11,000 [16]. These data do not challenge the validity of vaccination programs against SARS-CoV-2 in 2021, in view of the great severity of the disease. However, the relatively frequent occurrence and potential seriousness of vaccine-induced IMDs warrant the implementation of customized vaccination policies if the disease becomes more benign.

### 4.3. Types of Incriminated COVID-19 Vaccines 

The three types of available COVID-19 vaccines were implicated in IMDs. The higher frequency of BNT162b2-induced IMDs reflects the predominant use of this vaccine in 2021 in France. This is consistent with other case series [14,15,16,17], although other conditions, such as cerebral venous thrombosis and optic neuropathy reported to occur following COVID-19 vaccination have been markedly associated with the use of a specific vaccine [24,25]. Conversely, mRNA vaccines have inherent adjuvant properties that might have induced more adverse immune effects by generating Th17 inflammatory responses [26,27,28]. Finally, this and other studies indicate that IMDs can emerge at a variety of time points during vaccination schedules, typically after the first dose. 

### 4.4. Patient’s Age of Onset and Gender 

We found a slight female predominance (56%), and patients were mostly middle-aged or older. In the study by Watad et al., 15 of 27 (56%) patients were female and the mean age was 53 years (20–83 years) [14]. In the study by Kaulen et al., patients were mostly females (ratio 3.2:1) and had a median age of 50 years [16]. In a report on 19 immune-mediated events temporally associated with COVID-9 vaccination, only four patients (21%) were <50 years of age [15]. Regarding medical history, most (89%) patients in this study had new-onset IMDs, in agreement with other investigators [15,16,17], but not with the report by Watad et al., in which most COVID-19 vaccine-induced IMDs were flares of an underlying autoimmune or rheumatic disease [14]. Different settings, types of recruitment, and study designs may best explain the age-and-sex disparities among case series.

### 4.5. COVID-19 Vaccine-Induced Giant Cell Arteritis or Polymyalgia Rheumatica 

GCA/PMR was the main vaccine-related subset of IMDs, accounting for 59% of the cases. Notably, GCA represented 44% of the cases and 23% (11 of 47) of all new GCA cases in the Internal Medicine Department over the 17-month study. Including a patient with GCA following influenza vaccination, the proportion of post-vaccine GCA was 21% (e.g., 7 of 34 patients) in 2021, contrasting sharply with a mean of 3% annually for the previous decade. There are two explanations for this unexpectedly high proportion of post-vaccine GCA cases. First, awareness of the condition is increased [9], prompting inquiries during history taking. Second, mass COVID-19 vaccination of the regional older adult population not only increased the likelihood of a chance temporal association but also exposed a large number of at-risk subjects to the trigger. Other cases of GCA [16,25,29,30,31,32,33,34] or PMR [17,35,36] temporally associated with COVID-19 vaccination have been published, suggesting the association to be not casual and that post-vaccine onset of GCA/PMR is not an exceptional occurrence [9].

### 4.6. Other Types of COVID-19 Vaccine-Induced Vasculitis

In contrast to GCA/PMR, other vasculitides represented only 11% of COVID-19 vaccine-induced IMDs. Although vaccination is a known trigger of vasculitis [11], the most recent literature implicates COVID-19 vaccination, mostly in the form of case reports covering the whole spectrum of vasculitides, with heterogeneous clinical presentations and variable severities [37,38,39,40,41,42,43]. New-onset or relapsing ANCA-associated necrotizing vasculitides [37,38,39,40,41] or HSP [42,43] have been described. Significantly, a recent study using the WHO global safety database (Vigibase) found increased reporting of Behçet’s syndrome, microscopic polyangiitis, livedoid vasculopathy, and urticarial vasculitis following COVID-19 mRNA vaccination [44]. Whether this finding also applies to viral vector-based COVID-19 vaccines warrants further investigation.

### 4.7. COVID-19 Vaccine-Induced Immune-Mediated Necrotizing Myopathy or Acute Rhabdomyolysis

Immune-mediated necrotizing myopathy/rhabdomyolysis comprised 11% of the cases. A booster effect of COVID-19 vaccination on an underlying, silent, statin-induced IMNM in the patient positive for anti-HMG-CoA reductase antibody cannot be excluded. Other cases of inflammatory myositis or acute rhabdomyolysis of variable severity following SARS-CoV-2 vaccination have been reported [16,45,46,47,48,49,50,51]. Disease severity in the patients with acute muscle disorders ranged from a self-limited illness to a rapidly fatal rhabdomyolysis, despite intensive immunosuppressive therapy [46,50,51]. The relative paucity of reports of inflammatory myositis or acute rhabdomyolysis during the worldwide COVID-19 vaccination campaign is in line with the claim of no significant increase in the incidence of dermatomyositis or polymyositis following vaccinations of any type [52].

### 4.8. COVID-19 Vaccine-Induced Miscellaneous IMDs

Other IMDs in this study were miscellaneous, pointing to a ubiquitous mechanism of immune-response enhancement. Cases of ILD [53,54], RA [55,56], AOSD [57,58], and ITP [59,60] following COVID-19 vaccination have been reported, supporting the hypothesis of a causative link rather than a casual association. Interrogation of the French Pharmacovigilance Network yielded 123 ITP cases, the corresponding rate of de novo or relapsed reports per million COVID-19 vaccine doses being 1.69 (95% CI 1.42-201). In this study, the rate was highest with ChAdOx1-S, particularly after the first dose (60), whereas a previous study using the Vaccine Adverse Event Reporting System (VAERS) described 77 de novo ITPs, all after mRNA-based vaccines [59]. Our finding of two de novo ITPs but no severe relapse of a preexisting ITP is in agreement with previous reports that the latter is infrequent [59,60].

### 4.9. HLA-DR/DQ and COVID-19 Vaccine-Induced IMDs

The results of HLA-DRB1 typing in post-COVID vaccine IMDs did not yield a noticeable pattern, except for a high rate of DRB1*04 (54%) in GCA patients. We also observed a strong predominance, of unknown significance, of DQB1*03. Furthermore, the distribution of HLA-DRB1 alleles did not differ significantly among GCA/PMR subsets (e.g., post-COVID vaccine cases; post-influenza, or pneumococcal, vaccine cases; familial cases; typical cases). The origin of GCA is unknown but genetic and environmental factors are implicated [61]. Interestingly, in our inception GCA cohort, familial aggregation of GCA was detected in 2 of 12 (17%) patients with post-COVID-19 GCA and 4 of 13 (31%) patients with post-influenza or pneumococcal vaccine, but only 15 of 388 (4%) patients without a vaccine trigger. HLA-DRB1*04 is the main genetic determinant of GCA [62] and a risk factor for ASIA [8,63]. From our observations, unadjuvanted vaccines such as the currently marketed SARS-CoV-2 vaccines can induce GCA/PMR by acting as non-specific triggers in genetically predisposed subjects (notably those with HLA-DRB1*04) possibly by strongly activating Toll-like receptor signaling [64]. Nevertheless, a global pharmacovigilance study showed a lower relative risk of GCA or PMR following COVID-19 compared to influenza vaccination [65], consistent with the hypothesis that classical adjuvant-containing vaccines have a greater risk. Indeed, HLA-DRB1*01 was found only once, and no patient carried HLA-DQB1*01; both alleles are associated with ASIA [9,63]. The mixed HLA results highlight, therefore, the complex mechanisms underlying post-COVID vaccine IMDs.

### 4.10. Disease Severity in COVID-19 Vaccine-Induced IMDs

The severity of vaccine-induced IMDs is a concern. Contrasting with the favorable prognosis of patients in prior case series [14,15,16,17] and a recent review of post-COVID vaccination syndromes [66], in this study, few IMDs were self-limited and benign. On the contrary, 24% of the IMDs were complicated and most patients required prolonged glucocorticoid treatment, in combination with additional immunosuppressive or immunomodulatory agents in 40% of the cases. Post-COVID-19 vaccine IMDs can, therefore, be serious. Moreover, occurrence, or flare-up, of an IMD during the course of vaccination against COVID-19 can be diagnostically challenging, especially when the disease manifests as systemic symptoms such as fatigue, headache, and musculoskeletal pain soon after vaccination. Such a pattern of IMD onset can easily be mistaken for typical systemic adverse effects of vaccination, potentially delaying diagnosis.

### 4.11. Revaccination against COVID-19 after COVID-19 Vaccine-Induced IMDs

Revaccination against COVID-19 was acceptable. Indeed, 11 consenting patients were re-challenged with the same, or an alternate, vaccine, up to three additional shots, without significant harm in nine patients. However, the safety of re-challenging patients who experienced a COVID-19-induced IMD, and the optimal timing of re-challenge, warrants further research.

### 4.12. Limitations of the Study

This study has limitations that should be noted. The study design (hospital-based) precluded any calculation of the incidence of IMDs following COVID-19 vaccine. The association of COVID-19 vaccination with IMDs included may have been a result of chance, based on the small sample size and lack of evidence of a causal association [67]. In particular, de-challenge (i.e., evidence that the adverse event diminished, as would be expected if caused by the vaccine) was seen in only three cases, and re-challenge seldom resulted in disease relapse. Conversely, we performed the first teaching hospital, department-based study including all IMDs encountered during the period of mass vaccination, with exclusion of other causes, and extended follow-up. Moreover, this study is the first to address the relationship between COVID-19 vaccine-induced IMDs, especially GCA/PMR, and HLA-DR/DQ.

## 5. Conclusions

Our findings indicate that adults over 50 years of age may develop an IMD following COVID-19 vaccination. However, confirmation by other large-scale studies, especially focusing on the mass COVID-19 vaccination period, is warranted. We emphasize that such IMDs are rare and do not call into question the benefit of COVID-19 vaccination, which is overall safe and effective. By contrast, early revaccination after post-vaccine IMDs should be considered on an individual basis, according to disease severity, disease control, and patient acceptance. Finally, early re-challenge with a COVID-19 vaccine in patients with controlled IMDs is feasible.

## Figures and Tables

**Table 1:** Data of patients with immune-mediated diseases occurring, or relapsing, following COVID-19 vaccination.

Patient	Age/Sex	Condition	Vaccine	Dose	Delay (d)	Past History, Co-Morbid Conditions	Treatment for IMDs	Re-Challenge	Outcome
1	87/F	GCA	m-RNA (Moderna)	1st	7	Osteoarthritis	Pred 30 mg/d	Yes, twice	Recovered disease (13 mo, untreated for 4 mo)
2	82/F	GCA	m-RNA (Pfizer)	1st	10	Hypertension, osteoarthritis, gastric ulcer	Pred 40 mg/d	Yes, twice	Controlled disease (14 mo, Pred 3 mg/d)
3	70/F	GCA	viral vector (AstraZeneca)	1st	2	Diabetes, hypertension, hypercholesterolemia, gouty disease	Pred 40 mg/d, 2nd line Tocilizumab	No (declined)	Controlled disease (13 mo, Pred 7 mg/d)
4	90/F	GCA	m-RNA (Pfizer)	1st	3	MDS (5q-), past PMR, hypertension	MP 500 mg/d × 2, Pred 50 mg/d	Yes, once	Early lost to follow-up
5	80/M	GCA	m-RNA (Moderna)	2nd	3	None	MP 500 mg/d × 3, Pred 75 mg/d, 2nd line Tocilizumab	No (declined)	Relapsed disease (7 mo, Pred 15 mg/d)
6	65/F	GCA	m-RNA (Pfizer)	1st	3	Hypertension	Pred 50 mg/d	Yes, twice	Controlled disease (10 mo, Pred 6 mg/d)
7	59/F	GCA	m-RNA (Pfizer)	1st	4	Autoimmune neutropenia, breast carcinoma	Pred 60 mg/d, 2nd Tocilizumab	No (declined)	Multiple relapses upon short tapering Pred regimen (12 mo)
8	68/M	PMR, then GCA	viral vector (AstraZeneca)	2nd	20	Duodenal ulcer	Pred 50 mg/d	n.a.	Controlled disease (5 mo, Pred 10 mg/d)
9	64/M	GCA	m-RNA (Pfizer)	3rd	6	Familial aggregation (GCA in sister)	MP 500 mg/d × 3, Pred 80 mg/j Tocilizumab	n.a.	Controlled disease (3 mo, Pred 20 mg/d)
10	72/M	GCA	m-RNA (Pfizer)	3rd	5	Hypertension, diabetes, hypercholesterolemia	Pred 60 mg/d	n.a.	n.a. (recently diagnosed)
11	72/M	GCA (aortitis)	m-RNA (Pfizer)	3rd	1	Kidney stones	Pred 50 mg/d	n.a.	n.a. (recently diagnosed)
12	69/M	GCA	m-RNA (Pfizer)	3rd	2	Hypercholesterolemia	Pred 50 mg/d	n.a.	n.a. (recently diagnosed)
13	62/F	PMR	m-RNA (Pfizer)	2nd	6	Discogenic sciatica	Pred 30 mg/d, 2nd line Tocilizumab	Yes, once (Moderna)	Relapsed disease (7 mo, Pred 9 mg/d)
14	58/M	PMR	m-RNA (Pfizer)	2nd	2	Hypertension, obesity, pheochromocytoma, sleep apnea	Pred 40 mg/d	No (declined)	Controlled disease (9 mo, Pred 4 mg/d)
15	69/F	PMR	m-RNA (Pfizer)	1st	14	Hypertension, osteoarthritis, depression	Pred 20 mg/d	Yes, once (3rd dose planned)	Controlled disease (6 mo, Pred 5 mg/d)
16	77/M	PMR (late relapse)	m-RNA (Pfizer)	2nd	10	Hypertension, diabetes, prostatic carcinoma	Pred 20 mg/d	Yes (third dose)	Controlled disease (8 mo, Pred stopped for 2 mo)
17	64/M	AR	viral vector (AstraZeneca)	1st	13	Thyroid insufficiency, hypertriglyceridemia	MP 1 g/d × 3, Pred 2 mg/d, MTX, IVIG, CPM	No (prohibited)	Died of uncontrolled disease (2 mo)
18	59/F	IMNM	viral vector (AstraZeneca)	1st	11	Osteoarthritis	MP 1 g/d × 3 + IVIG, Pred 60 mg/d	No (declined)	Recovered (13 mo, no treatment for 12 mo)
19	62/M	IMNM	m-RNA (Pfizer)	3rd	7	Hypertension, sleep apnea, nocardiosis, benign ampulloma	Pred 80 mg/d, IVIgG	n.a.	Satisfactory short-term response
20	57/F	AAV (EGPA)	viral vector (AstraZeneca)	1st	14	Allergic asthma	MP 1 g/d × 3, Pred 70 mg/d, RTX	Yes (twice, m-RNA based)	Limited disease relapse (11 mo, pred 5 mg/d + RTX 500 mg every 6 mo)
21	50/F	SHP (late relapse)	m-RNA (Pfizer)	1st	2	Pediatric SHP	Colchicine, pregabaline	Yes, twice	Relapsed disease (10 mo, upon colchicine discontinuation)
22	53/M	SHP (digestive involvement)	m-RNA (Pfizer)	1st	8	None	MP 1000 Mg/d × 4, Pred 80 mg/d, colchicine	No (declined)	Controlled disease (12 mo, Pred 2.5 mg/d + colchicine)
23	61/F	Anti-synthetase (PL7+)	m-RNA (Pfizer)	2nd	4	Hypothyroidism	Pred, 60 mg/d, 2nd line tacrolimus	n.a.	Poorly controlled disease (5 mo, Pred 15 mg/d)
24	69/F	AOSD relapse	m-RNA (Pfizer)	3rd	14	Hypertension	Pred 20 mg/d, oral methotrexate	n.a.	Relapsed disease (4 mo, pred 10 mg/d, methotrexate resumed)
25	73/F	ITP	viral vector (AstraZeneca)	1st	14	Hypertension, hypercholesterolemia, COPD, atherosclerotic peripheral disease	Dex 40 mg/d × 4, Pred 60 mg/d	Yes, twice (m-RNA based)	Recovered disease (11 mo)
26	71/M	ITP	viral vector (AstraZeneca)	1st	4	Sleep apnea, psoriasis, benign prostate hypertrophy	Dex 40 mg/d × 4, Pred 70 mg/d, TRA	No (declined)	Failure-partial control under TRA (11 mo)
27	57/F	RA (late relapse)	m-RNA (Pfizer)		2	Sleep apnea	methotrexate	n.a.	n.a. (recently diagnosed)

GCA, giant cell arteritis. AR, acute rhabdomyolysis. IMNM, immune-mediated necrotizing myopathy. AAV, ANCA-associated (systemic) vasculitis. EGPA, eosinophilic granulomatosis with polyangiitis. SHP, Schönlein-Henoch purpura. AOSD, adult-onset Still’s disease. RA, rheumatoid arthritis. ITP, immunologic (idiopathic) thrombocytopenic purpura. MDS, myelodysplastic syndrome. PMR, polymyalgia rheumatica. BPH, benign prostate hypertrophy. COPD, chronic obstructive pulmonary disease. Pred, prednisone. MP, methylprednisolone (pulse high dose). MTX, methotrexate. IVIG, intravenous immunoglobulins. CPM, cyclophosphamide. Dex, dexamethasone. TRA, thrombopoietin receptor agonist.

**Table 2:** DQB1 typing of 20 patients with post-COVID-19 vaccine IMDs.

Patient	Diagnosis	HLA DRB1/DQB1 Typing
Case 1	GCA	DRB1*04:02/*08:01; DQB1*03:02/*04:02
Case 2	GCA	DRB1*07:01/*11:04; DQB1*02:02/*03:01
Case 3	GCA	DRB1*07:01/*07:01; DQB1*02:01/*02:02
Case 5	GCA	DRB1*03/*04; DQB1*02/*03
Case 6	GCA	DRB1*04:04/*11:01; DQB1*03:01/*03:02
Case 7	GCA	DRB1*04:01/*15:01; DQB1*03:01/*06:02
Case 8	GCA	DRB1*04:03/*15:02; DQB1*03:02/*06:01
Case 9	GCA	DRB1*03:01/*04:01; DQB1*02:01/*03:01
Case 10	GCA	DRB1*07:01/*13:01; DQB1*02:01/*06:03
Case 11	GCA	DRB1*04:02/*15:01; DQB1*03:02/*06:02
Case 12	GCA	DRB1*01:02/*11:02; DQB1*03:01/*05:01
Case 13	PMR	DRB1*03/*12; DQB1*02/*03
Case 14	PMR	DRB1*11:01/*16:01; DQB1*03:01/*05:02
Case 16	PMR	DRB1*13/*14; DQB1*03/*05
Case 17	AR	DRB1*07:01/*08:01/DQB1*02:02/*04:02
Case 18	IMNM	DRB1*11:04/*16:01; DQB1*03:01/*05:02
Case 19	IMNM	DRB1*04:01/*11:01; DQB1*--/*--
Case 23	ASS (PL7+)	DRB1*01:01/*17:01; DQB1*02:02/*05:01
Case 24	SHP	DRB1*07:01/*11:04; DQB1*03:01/*06:01
Case 27	RA	DRB1*01:01/*13:01; DQB1*05:01/*06:01

GCA, giant cell arteritis. PMR, polymyalgia rheumatica. AR., acute rhabdomyolysis. ASS, antisynthetase syndrome. SHP, Schönlein-Henoch purpura. RA, rheumatoid arthritis.

**Table 3:** Typing in 58 patients—50 with GCA and 8 with PMR, under various circumstances of onset.

HLA-DRB1 Allele	Post-COVID-19 Vaccine Cases N = 14 *n* (%) *	Post-Other-Vaccine Cases N = 11 *n* (%) ^†^	Familial Cases N = 17 *n* (%) ^§^	Other Cases N = 16 *n* (%) ^¥^	*p*-Value ^¶^
DRB1*01	1 (7)	2 (18)	0 (0)	2 (12.5)	0.30
DRB1*03	3 (21)	2 (18)	1 (6)	2 (12.5)	0.63
DRB1*04	7 (50)	4 (36)	10 (59)	10 (62.5)	0.58
DRB1*07	3 (21)	3 (27)	7 (41)	4 (25)	0.68
DRB1*08	1 (7)	1 (9)	0 (0)	0 (0)	0.34
DRB1*09	0 (0)	1 (9)	0 (0)	0 (0)	0.19
DRB1*10	0 (0)	1 (9)	0 (0)	0 (0)	0.19
DRB1*11	5 (36)	2 (18)	5 (29)	4 (25)	0.83
DRB1*12	1 (7)	0 (0)	0 (0)	0 (0)	0.43
DRB1*13	2 (14)	5 (45)	5 (31)	5 (31)	0.40
DRB1*15	3 (21)	1 (9)	4 (23.5)	3 (19)	0.86
DRB1*16	1 (7)	0 (0)	0 (0)	0 (0)	0.43

* Eleven GCA and three PMR. ^†^ Seven GCA and four PMR. ^§^ Sixteen GCA and one PMR. ^¥^ GCA only. ^¶^ Post-COVID vaccine cases vs. post-other-vaccine cases vs. familial cases vs. other cases.

## Data Availability

Data is contained within the article.
